# Impact of COVID-19 on Cardiothoracic Surgery: Experience of Alexandria (Egypt) Main University Hospital

**DOI:** 10.21470/1678-9741-2020-0551

**Published:** 2022

**Authors:** Hanan M. Hemead, Mohamed H. Elsayed, Wael Hassanein

**Affiliations:** 1 Department of Cardiothoracic Surgery, Alexandria University of Medicine, Alexandria, Egypt.

**Keywords:** Cardiac Surgical Procedures, Thoracic Surgery, Coronavirus, Pandemics, Patient Care Team, Egypt

## Abstract

**Introduction:**

The current coronavirus pandemic has greatly strained the limited resources that had previously maintained the sustainability of the high-cost cardiothoracic surgeries in low-income countries like Egypt.

**Methods:**

Hospital databases and patients’ records were reviewed to evaluate the impact of the pandemic on the workflow and waiting lists. Postoperative patients were contacted by telephone for follow-up, as well as preoperative patients whose operations were cancelled. Regular virtual meetings were held, and residents were asked to discuss the stresses, challenges, and their suggestions for the gradual resumption of services. Residents’ logbooks were evaluated to assess the disruption of the surgical exposure compared to 2019.

**Results:**

While thoracic surgeries have continued to thrive, cardiac surgeries have witnessed the worst consequences, including cancellation of all surgeries, expansion of waiting lists, patients’ non-compliance with follow-up, and impaired surgical exposure of junior residents.

**Conclusion:**

The gradual recovery of cardiac surgery services in Alexandria (Egypt) is being carefully planned, taking into consideration the backlog of cases and the shortage of screening kits. Careful tiering and triaging of patients by a multidisciplinary team, as well as seeking alternative assessment tools for trainees, are the main lines of our action plan.

**Table:** 

Abbreviations, acronyms & symbols	
CABG	= Coronary artery bypass graft
COVID-19	= Coronavirus disease 2019
ICU	= Intensive care unit
MDT	= Multidisciplinary team
PCR	= Polymerase chain reaction

## INTRODUCTION

The coronavirus disease 2019 (COVID-19), caused by the novel severe acute respiratory syndrome coronavirus 2 (or SARS-CoV-2), has become the worst global health emergency. The pandemic has had grave consequences on health care systems worldwide and profound impacts on low-income and developing countries with poor health and financial infrastructure^[1,2]^. The pandemic also had devastating effects on the workflow of cardiac and thoracic surgeries worldwide. Some centers needed to cancel all elective surgeries or significantly reduce the case volume^[3,4]^.

In Egypt, the situation is more challenging as the existing critical care system suffers from constrained resources^[5]^. Early in the pandemic, the Egyptian Ministry of Health and Population transformed non-busy hospitals into isolation centers. To minimize the spread of COVID-19, health care workers have been rotated every two consecutive weeks of work in these facilities and then kept in home quarantine for another two weeks. Later in the pandemic, isolation wards were designated in some governmental hospitals, where meticulous infection control measures were applied. The official action plan included decreasing both inpatient admissions and outpatient visits, as well as cancellations of elective surgeries. Due to the exponential rise of caseload and the increasing financial burden, nasopharyngeal swabs were restricted to highly suspected and severely symptomatic cases requiring hospital admission.

The Department of Cardiothoracic Surgery in the Alexandria Main University Hospital is one of the busiest cardiothoracic centers in Egypt. It serves patients in the metropolitan area of Alexandria and nearby towns and cities. Since the declaration of strict shutdown, all elective cardiac surgeries have been postponed. Considering the huge population served by our institute and the vulnerability of this category of patients, prompt actions were needed to ensure the delivery of safe and optimal care.

Surgeons have been devastatingly affected by the pandemic. They were assigned to care for severely ill patients in intensive care units (ICU), which exposed them to a medical specialty far from their main training and objectives. The quality of surgical training is threatened, due to the decreased number of operations.

As there is an international and national consensus on relaxing the restrictions and on the gradual restoration of health services, a careful action plan must be contemplated, based on the national current situation and backlog of cases awaiting their surgeries. The current challenges facing our institute are herein presented, as well as plans for the gradual recovery of services.

## METHODS

Hospital databases were used to retrieve data about the impact on the flow of cases during the pandemic (2020), compared to the same period in 2019. Data were collected from the thoracic, cardiac, and trauma departments. Patients’ records and waiting lists were used for the follow-up of postoperative patients, who were reluctant to attend the clinics, and for assessment of patients awaiting their surgeries. Weekly virtual meetings were held by the surgical team to discuss challenges and plans. Residents’ logbooks were evaluated to assess the disruption of surgical exposure during the pandemic.

## RESULTS

### Adult Cardiac Surgery

The average annual case volume of adult cardiac surgery in the institute is 300-350 cases. On March 21^st^, 2020, the hospital staff were instructed to postpone new admissions from cardiac surgery outpatient clinics and to perform surgeries exclusively for patients currently admitted and emergencies. On June 16^th^, 2020, we admitted an emergency case of critical left main stenosis. As nasopharyngeal swabs and polymerase chain reaction (PCR) testing were unavailable, full blood count, inflammatory markers, and chest radiographs were used as screening tests. Postoperatively, the patient developed heparin-induced thrombocytopenia, dyspnea, fever, cough, and prolonged air leak from the chest drains. Computed tomography of the chest was consistent with COVID-19 indications. The patient was transferred to an isolation center after the swab turned positive for coronavirus and was discharged home two weeks later. Since March 21^st^, 2020, until the present time, all elective cardiac surgery cases were postponed. After admission of the last emergency case in June 2020, all open-heart theatres and cardiac ICU were transformed into isolation wards. Emergency cases were referred to the largest tertiary care center in Cairo City (the capital). The numbers of cardiac surgeries performed during the pandemic, compared to the same period in 2019, are shown in Table 1.

**Table 1:** Number of cardiac surgeries and types of interventions performed in the Department of Cardiothoracic Surgery in Alexandria Main University Hospital during the period from March 21^st^ to October 1^st^, 2020, compared to the same period in the previous year.

Intervention	March 21^st^ - October 1^st^, 2020 (number of cases)	March 21^st^ - October 1^st^, 2019 (number of cases)
Isolated coronary artery bypass graft (CABG)	6	80
Isolated valvular replacement (one or double valve replacement)	4	41
Combined CABG and valve replacement	2	12
Aortic surgeries	0	3
Pectoral flap for gapped sternotomy wound (post CABG)	2	0
Others	0	6*

* Pericardiectomy, excision of left atrial myxoma

Our unit is the only governmental center (belonging to the public health sector) in Alexandria performing cardiac surgery. Three smaller private centers operate a much lower annual case volume of less complex cardiac surgeries, due to the high expense and limited resources. These centers reported a 25% to 50% reduction in the case volume during the pandemic.

Patients were contacted by telephone for follow-up. One patient with a prosthetic valve, who used to have troubles with warfarin dose adjustment, was lost for follow-up. He presented with warfarin toxicity and tamponading effusion and had subxiphoid pericardiostomy.

Two postoperative patients had deep mediastinal wound infections and were admitted for administration of intravenous antibiotics, according to culture and sensitivity tests. A negative suction system was applied, and they were discharged home. They updated the unit with weekly images of their wounds to assess the healing progress.

Preoperative patients on the waiting lists, whose operations were cancelled, are regularly contacted. Patients with critical pathologies, such as critical coronary disease and severe aortic stenosis, were referred to the largest tertiary care center in Cairo for emergency admission. Unfortunately, we traced 50 patients with coronary stenosis and five patients with valvular diseases who died since the cancellation of elective surgeries. Most of them resisted having surgeries during the peak of the pandemic, and the remainder died during the process of referral.

### Thoracic Surgery

The annual case volume of thoracic surgeries in the center is between 350 and 400 cases. Full blood count and computed tomography of the chest were performed preoperatively. Suspected cases were asked to provide a negative nasopharyngeal swab. Thoracic surgeries were continued to be performed, as the operating theatres and intensive care beds were not transformed into isolation units. During the peak of the pandemic, selective admission of cases was made to ensure the prioritization of patients with potentially urgent conditions. To ensure social distancing, admissions from outpatient clinics were significantly reduced. The numbers of thoracic surgeries performed during the pandemic, compared to the same period in 2019, are shown in Table 2.

**Table 2:** Number of thoracic surgeries and types of interventions performed in the Department of Cardiothoracic Surgery in Alexandria Main University Hospital during the period from March 21^st^ to October 1^st^, 2020, compared to the same period in the previous year.

Intervention	March 21^st^ - October 21^st^, 2020 (number of cases)	March 21^st^ - October 21^st^, 2019 (number of cases)
Lung resection; lobectomy/wedge	2	10
Lymph node biopsy	5	11
Pleural surgeries (biopsy/decortication)	14	33
Esophageal surgeries	1	7
Tracheal and bronchial surgeries	1	1
Endoscopic procedures (esophagoscopy/bronchoscopy)	7	45
Lung reduction surgeries (e.g., bullectomy)	6	2
Thymectomy (for myasthenia gravis)	2	3
Feeding gastrostomy	1	4
Diaphragmatic plication	0	3
Thoracoscopic sympathectomy	10	20
Chest wall surgeries (rib resection/abscess evacuation, de-wiring/flaps)	2	22
Others	0	6*
Reoperation	3	4

* Pleuropericardial window, mediastinal masses

### Trauma Cases

During the strict national lockdown, the number of cases admitted to the emergency room for chest trauma was significantly low. Three cases with penetrating chest trauma were admitted for exploration, due to hemodynamic instability on presentation. All patients had an uncomplicated postoperative course. In order to shorten the duration of hospital stay, when stable, patients with spontaneous pneumothoraxes and prolonged air leak were offered to use the Heimlich valve and follow up in the outpatient clinics.

### Impact on Surgical Training

In addition to the weekly virtual meetings in which residents gave their feedbacks, logbooks were used as an objective tool to evaluate the impact of the pandemic on surgical training. In our center, the training period is five years, and two residents are enrolled in the program. Table 3 shows the interventions completed by a four-year resident compared to the same period in 2019.

**Table 3:** Number of procedures performed by a four-year resident during the pandemic compared to the same period during the previous year (data collected from the resident’s logbook).

Intervention	March 21^st^ - October 1^st^, 2020		March 21^st^ - October 1^st^, 2019	
	Number of cases	Level of participation*	Number of cases	Level of participation*
Saphenous vein harvesting	5	A	115	A
Cannulation for bypass	1	B	15	B
Median sternotomy	2	A	96	A
Sternal closure	2	A	96	A
Thoracotomy	3	A	35	A
Pleural decortication	1	C	5	B

* A=the procedure was done by the trainee without the main surgeon assisting; B=trainee, assisted by a senior resident; C=trainee is the first assistant in the procedure.

## DISCUSSION

Curtailing all elective cases to preserve critical care beds was mandatory. However, it is sometimes difficult to precisely define true “elective” cases in cardiac surgery, owing to the progressive nature of the underlying etiology. It is not uncommon that a so-called elective case becomes an emergency^[6-11]^, which significantly increases mortality rate. Death during awaiting a scheduled cardiac surgery was frequently reported and unfortunately witnessed in the center during the lockdown^[12-15]^. Though all released recommendations emphasized screening swabs and PCR, this might be impractical in low-income countries due to the high cost. To decrease the financial impact, stratification of patients can be done according to the recommendations of the American Heart Association^[16]^. Patients with a high pretest probability will have a PCR test. Subjects with low susceptibility will have to undergo chest imaging, full blood count, and inflammatory markers test, such as C-reactive protein and serum ferritin. Multidisciplinary team (MDT) meetings are arranged to discuss each individual case and to prioritize admission, especially for cases with an expected lengthy stay in ICU due to the complexity of procedure and preexistent comorbidities. There is a growing trend to refer more cases to percutaneous coronary intervention and transcatheter valve replacement during the peak of the pandemic. Therefore, MDT meetings will have to make thoughtful casewise decisions.

The database is currently updated to anticipate the impact of COVID-19 on the waiting list. To prevent overcrowding, selected cases, according to MDT meetings, are contacted. Patients with low susceptibility and normal lab tests will be asked to come one day prior to surgery for anesthesiologists’ assessment, blood group matching, and computed tomography of the chest.

Surgical teams have been regularly educated on infection control measures. Segregation of members of the surgical team into smaller teams is a valid option to keep the workflow, if one of the members becomes ill or isolated^[17]^.

The negative consequences on surgical training should be addressed and dealt with thoroughly at the level of the global surgical community. During the lockdown, several sessions were arranged for echocardiography training, biostatistics, scientific writing, and case-based discussions by senior members in the department. Junior residents were satisfied to improve other aspects of surgical training than hand skills. However, senior residents were deprived from the opportunity of polishing their surgical skills by the end of their residency. As the logbooks are the widely acceptable objective method of assessment, other tools should be discussed later. The solutions adopted in our centers are the extension of the training period for a few months and the assignment of more procedures to final-year residents when elective surgeries are resumed.

Despite that telehealth services are not widely acceptable by the population, it was found that postoperative patients were increasingly satisfied with telephone checkup appointments and direct message contact. This attitude is encouraging us to develop a regular telehealth service in the near future.

## CONCLUSION

COVID 19 has posed significant implications for cardiac and thoracic patients, the integrity of cardiothoracic centers, and quality of training. These negative consequences were more pronounced in the strained health systems of developing countries. Stepwise restoration of services should be carefully established and guided by MDT meetings and case-dependent decisions. The extended effect on surgical training should be evaluated, and different assessment tools can be discussed. As we remain uncertain of the magnitude of COVID-19 in the future, we need to remain open-minded to the major challenges, and willing to implement dynamic adaptive policies.

**Table:** 

Authors' roles & responsibilities	
HMH	Substantial contributions to the conception and design of the work; drafting the work and revising it; final approval of the version to be published
MHE	Substantial contributions to the conception and design of the work; and the analysis and interpretation of data for the work; final approval of the version to be published
WH	Substantial contributions to the conception and design of the work; and the analysis and interpretation of data for the work; revising the work critically for important intellectual content; final approval of the version to be published
